# Prevalence and Treatment Outcomes of Syphilis among People with Human Immunodeficiency Virus (HIV) Engaging in High-Risk Sexual Behavior: Real World Data from Northern Greece, 2019–2022

**DOI:** 10.3390/microorganisms12071256

**Published:** 2024-06-21

**Authors:** Sideris Nanoudis, Dimitrios Pilalas, Theologia Tziovanaki, Margarita Constanti, Konstantinos Markakis, Konstantinos Pagioulas, Eleni Papantoniou, Konstantina Kapiki, Theofilos Chrysanthidis, Panagiotis Kollaras, Symeon Metallidis, Olga Tsachouridou

**Affiliations:** Infectious Diseases Unit, 1st Internal Medicine Department, AHEPA University Hospital, School of Medicine, Aristotle University of Thessaloniki, 554 36 Thessaloniki, Greece; sidnanoudis@yahoo.gr (S.N.); dpilalas@auth.gr (D.P.); theologia1996@gmail.com (T.T.); margaritaaconstanti@gmail.com (M.C.); conmark@windowslive.com (K.M.); kpagioulas@gmail.com (K.P.); elenispap@yahoo.gr (E.P.); kwnnakap@gmail.com (K.K.); teochriss@yahoo.co.uk (T.C.); metallidissimeon@yahoo.gr (S.M.)

**Keywords:** syphilis, people with human immunodeficiency virus, high-risk sexual behavior, men who have sex with men, benzathine penicillin G, doxycycline

## Abstract

In this study, we aimed to assess the prevalence of syphilis among people with human immunodeficiency virus (HIV; PWH) engaging in high-risk sexual behavior, determine the stage of syphilis, and evaluate treatment efficacy. A retrospective single-center cohort study was conducted at the AHEPA University General Hospital of Thessaloniki, focusing on PWH at high risk for sexually transmitted infections (STIs) attending outpatient care from January 2019 to December 2022. Sociodemographic and clinical data were collected, incident syphilis rates were identified, associations with HIV-related characteristics were explored, and the treatment response was assessed. Among 991 participants, 94 PWH were diagnosed with syphilis, representing 9.4% of the cohort. Incident syphilis cases experienced a decrease in the early COVID-19 era compared to 2019, followed by a gradual increase leading up to 2022. The majority of syphilis cases were asymptomatic latent syphilis (71.1%). Men who have sex with men (MSM) and younger individuals exhibited higher rates of co-infection during the study period. No significant association was found between incident syphilis and HIV-related factors. Most syphilis cases (86%) were treated with benzathine penicillin G (BPG). Treatment with BPG and doxycycline showed an increased success rate (96.7% vs. 92.9%), with no statistically significant difference observed between them (*p* = 0.438). This study highlights the alarming incidence of syphilis among PWH engaging in high-risk sexual behavior, particularly among younger MSM. BPG remains effective, and alternative regimens like doxycycline show promise, especially in settings with penicillin shortages or patient allergies.

## 1. Introduction

Syphilis and human immunodeficiency virus (HIV), two of the most prevalent sexually transmitted infections (STIs) worldwide, continue to pose significant public health challenges [1,2,3]. Individually, each infection carries its own set of complexities, yet when co-existing within the same individual, they can amplify the severity of their respective clinical outcomes and transmission risks [4]. The co-occurrence of syphilis and HIV presents unique challenges due to the complex interaction between these two infections [5]. Understanding the interplay between syphilis and HIV is crucial for effective prevention, diagnosis, and management strategies [5,6]. Syphilis can increase the risk of HIV acquisition and transmission through genital ulcers, while HIV infection and antiretroviral therapy (ART) can alter the natural history and clinical presentation of syphilis [7,8,9,10]. Furthermore, the immunosuppressive effects of HIV can complicate the diagnosis and treatment of syphilis, leading to atypical manifestations and potential treatment failures [10,11,12]. Cutaneous secondary syphilis, known as the “great imitator”, may present in atypical forms in people with HIV (PWH), such as lupus vulgaris and purulent dermatitis [13,14]. Addressing the threat of drug resistance, syphilis requires a multifaceted approach, including enhanced surveillance, antimicrobial stewardship, and consideration of alternative treatment regimens [15,16].

The observed rates of syphilis have been on a substantial rise globally over the last two decades [17]. The resurgence of syphilis rates since 2000 might be attributed to several interconnected factors, including changes in sexual behavior and decreased awareness and fear of HIV and acquired immunodeficiency syndrome (AIDS) [18]. With the advent of ART for HIV/AIDS in the late 20th century, the perception of HIV/AIDS shifted from a terminal illness to a manageable chronic condition [19]. Prolonged survival from HIV infection, the concept of U = U (undetectable = untransmittable), and the initiation of PrEP (Pre-Exposure Prophylaxis) may have led to increased risky sexual behaviors, including higher rates of unprotected condomless sex, multiple sexual partners, and the rise of anonymous sexual encounters facilitated by dating apps and online platforms [18,19,20]. Certain population groups are disproportionately affected by syphilis, including men who have sex with men (MSM), transgender individuals, PWH, and people injecting drugs [4,17,21]. However, sporadic outbreaks of syphilis have also been reported among pregnant women, commercial sex workers, and heterosexual individuals experiencing socioeconomic disadvantage [1,2].

In 2022, there was a crude notification rate of 8.5 syphilis cases per 100,000 people in the European Union (EU) region, representing a 41% increase compared to 2018 and reaching an all-time high since 2013, according to the annual epidemiological surveillance report from European Centre for Disease Prevention and Control (ECDC) [2]. Three-quarters of cases were reported in MSM, showing a steep increase in this transmission group during the last decade [2]. A total of 26% of the MSM cases with information on HIV status were people with HIV co-infection. Greece was among the countries with the highest rates of confirmed syphilis cases in the EU (≥7 cases per 100,000 people) and also the highest observed male-to-female ratio (above 10:1) [2].

Penicillin is the reference standard in syphilis treatment; however, it is often commercially unavailable in Greece, and healthcare providers should consider alternative treatment options [22]. In most cases, doxycycline is used as a substitute antibiotic based on the stage of syphilis and individual patient factors [22]. The aim of this study is to investigate the rates of incident syphilis in PWH with high-risk sexual behavior during the prepandemic period and the COVID-19 era, delineate its stages (primary, secondary, latent, tertiary, neurosyphilis), assess sociodemographic factors influencing its occurrence, and examine treatment options and efficacy utilized in affected individuals.

## 2. Materials and Methods

### 2.1. Study Design

This was a retrospective, single-center cohort study conducted at AHEPA University General Hospital of Thessaloniki. Our Department of Infectious Diseases currently provides follow-up care every 6 to 8 months for approximately 1800 PWH. Serological tests for syphilis are routinely performed on individuals engaging in high-risk sexual behavior, including those with a history of syphilis, other STIs, multiple sexual partners, and those who do not consistently use protection during sex. PWH exhibiting high-risk sexual behavior who attended outpatient clinical care between 1 January 2019 and 31 December 2022 were enrolled in the study.

Sociodemographic and epidemiological data were obtained through the electronic medical record system and clinical visits. This information included gender, age, ethnicity, mode of HIV transmission, duration of HIV infection, duration of ART, and history of syphilis. HIV RNA plasma levels were quantified using real-time PCR, with the lower limit of detection set at 20 copies/mL, and CD4+ T-cell counts were determined by flow cytometry. Suppressed viral load was considered as HIV RNA < 50c/mL. The serological tests for syphilis included a treponemal test using chemiluminescence immunoassay (CIA) and a non-treponemal test using Rapid Plasma Reagin test (RPR). We used the reverse syphilis screening algorithm, with the primary screening test performed using the CIA test. In case of positive CIA test, quantitative RPR test was used to determine serological activity of syphilis and monitor the effect of treatment in accordance with 2024 Centers for Disease Control and Prevention (CDC) laboratory recommendations for syphilis testing, 2021 CDC treatment guidelines on sexually transmitted infections, and 2020 European guideline on the management of syphilis [22,23,24].

This study was conducted in accordance with the Declaration of Helsinki, and the protocol was approved by the local Ethics Committee under protocol number 21482/24.

### 2.2. Definition of Incident Syphilis and Stages

Incident syphilis was defined by a new seroconversion of CIA test or a fourfold or greater increase in RPR test titers in a person with known previous history of syphilis. The definition of syphilis stages was established based on clinical findings. Cerebrospinal fluid evaluation was performed only in persons with clinical signs of neurosyphilis (e.g., cranial nerve dysfunction, meningitis) according to recent guidelines, and further ocular and otic examination was warranted for those with ocular symptoms and auditory abnormalities, respectively [22]. We defined latent syphilis as the presence of seroreactivity with no clinical evidence of primary, secondary, or tertiary disease. Early latent syphilis was considered when infection occurred within the last 12 months, as documented by a negative treponemal test within 1 year of a syphilis diagnosis or a fourfold or greater increase in RPR titers within 1 year of previous testing. Late latent syphilis (>1 year’s duration) was assumed in an asymptomatic individual with positive syphilis serology if they did not meet the criteria for early latent syphilis.

### 2.3. Syphilis Treatment

PWH diagnosed with primary, secondary, and early latent syphilis were treated with benzathine penicillin G (BPG) 2.4 million units intramuscularly (IM) in a single dose as the first-line therapy, whereas participants diagnosed with late latent syphilis received 3 doses of BPG 2.4 million units IM at 1-week intervals. In case of penicillin shortage or history of allergy or intolerance, we used doxycycline as an alternative regimen. The dosage of doxycycline was 100 mg orally twice a day for 14 days for the treatment of primary, secondary, and early latent syphilis and 100 mg orally twice a day for 28 days for the treatment of late latent syphilis. Intravenous ceftriaxone was used for the treatment of neurosyphilis due to penicillin allergy.

### 2.4. Treatment Response

Serological evaluation was performed at 6 and 12 months after treatment. An adequate therapeutic response was defined by the complete loss of nontreponemal antibodies or a fourfold decline in RPR titers, equivalent to a change of two dilutions, at 12th month. Definition of serological nonresponse and treatment failure included a lack of a fourfold decrease in RPR titers at 12 months after treatment and a sustained (>2 weeks) fourfold increase in nontreponemal test titers at any time after treatment or recurrence or persistence of symptoms, respectively. In instances of treatment failure, participants were queried regarding the potential for syphilis reinfection and were subsequently excluded from the study if they engaged in risky sexual behavior.

### 2.5. Statistical Analysis

Quantitative data were checked for normality using the Shapiro–Wilk test and are reported as median and interquartile range (IQR). Qualitative data are presented as numbers and percentages. Fisher’s exact test and Pearson’s chi-square test were employed to assess associations between categorical variables, as appropriate. The non-parametric Mann–Whitney U test and Kruskal–Wallis test were utilized for comparisons between two or more groups, respectively. Post hoc testing with Dunn’s test was conducted for multiple comparisons. Bivariate and multivariable logistic regression analyses were used to predict new syphilis diagnoses. Factors with a *p*-value ≤ 0.25 in the univariable analysis were included in the multivariable analysis. The level of significance was set at a *p*-value of less than 0.05. Data were analyzed using IBM SPSS Statistics 26.0 (IBM Corp., Armonk, NY, USA).

## 3. Results

### 3.1. Baseline Characteristics of Study Participants

A total of 1896 syphilis tests were conducted on 991 PWH during the study period. The vast majority of the study participants were Greek males (Table 1). The main mode of transmission involved MSM (64.7%). The median age at baseline was 41.3 (34.3–49.7) years, and the median duration of HIV infection was 4.7 (0.3–10.3) years. A total of 320 (32.3%) participants had a history of previous syphilis infection (48.8% of the tests performed), as indicated by positive treponemal antibody testing. The MSM population exhibited the highest rate of positive syphilis history (41.3%) compared to the other transmission risk groups (15.6%) (χ^2^(1) = 69.3, *p* < 0.001, Pearson’s chi-square test). A total of 249 participants were tested for syphilis after being recently diagnosed with HIV infection and during their initial referral to our Infectious Diseases unit. The median CD4+ T-cell count was 670 (483–882) cells/mm^3^, and HIV viral load was undetectable (<50 copies/mL) in 81.5% of the syphilis tests conducted.

### 3.2. Rate of Syphilis Testing

Throughout the entire follow-up period, 436 (44%), 240 (24.2%), and 123 (12.4%) participants were tested at least two, three, and four times for syphilis, respectively. Syphilis testing was conducted at least twice for 54.9% of individuals in the MSM group, 35.4% of PWH with unknown transmission risk, 22.9% of heterosexuals, and 17.5% of PWID. There was a progressive rise in the frequency of syphilis testing over the years of the study (Table 2). Men underwent syphilis testing per person at a higher rate compared to women (U = 27,700, *p* < 0.001). Greek PWH were tested more frequently than non-Greek PWH (U = 35,821, *p* < 0.001). Compared with heterosexuals, PWID, and those with unknown transmission risk, MSM had a statistically significant higher rate of syphilis testing (H(3) = 94.5, *p* < 0.001). The rates of testing were lower among PWID. No variation was noted in the frequency of syphilis testing based on age groups (H(4) = 5.1, *p* < 0.282).

### 3.3. Rate of Incident Syphilis

**Figure 1:**
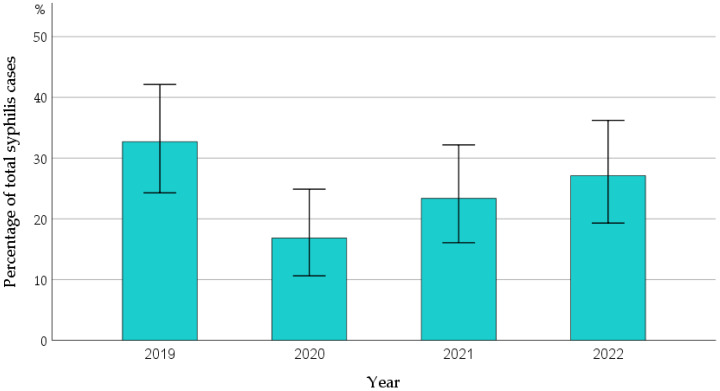
Rates of confirmed syphilis cases per year during the study period.

A total of 107 incident syphilis cases (5.6% of tests), all in males, were identified in 94 PWH (9.4% of study participants); 82 of them had one, 11 had two, and 1 had three episodes of syphilis during the study period. The occurrence of syphilis exhibited a decline in 2020 compared to 2019, followed by a gradual rise until 2022 (Table 2, Figure 1). A total of 99 (92.5%) of the syphilis episodes occurred in Greek people and 97 (90.5%) in MSM. The median age of the newly diagnosed syphilis cases was 37.6 (31.8–46.8) years. Seventeen participants (6.8%) with recently diagnosed HIV infection were also found to have syphilis coinfection during their initial clinical visit.

### 3.4. Sociodemographic and HIV-Related Predictors of Incident Syphilis Cases

Compared to other transmission risk groups, MSM were more likely to be co-infected with syphilis at the time of HIV diagnosis (OR: 7.02, 95%CI: 1.57, 31.35, *p* = 0.011). Similarly, MSM were more likely to contract syphilis during the study period compared to other modes of transmission (Table 3). There was a positive association between younger age and the likelihood of syphilis diagnosis. Among age groups, PWH aged 30–39 had the highest rate of incident syphilis, while PWH aged over 60 years had the lowest. The duration of HIV infection, duration of ART, nationality, CD4+ T-cell count, detectable HIV viral load, and history of previous syphilis infection did not show any association with the development of syphilis. Excluding male sex, MSM were the only independent predictors of the occurrence of new syphilis cases in the multivariable logistic regression model (χ^2^(8) = 33.6, *p* < 0.001) (Table 3).

### 3.5. Stages of Syphilis

The majority (71.1%) of incident syphilis cases were classified as latent syphilis, 18.7% were secondary syphilis, 9.3% were primary syphilis, and one case (0.9%) of neurosyphilis was observed (Table 4). Out of the 76 cases of asymptomatic latent syphilis, 51 (67%) were classified as late latent syphilis, with the remaining cases categorized as early latent syphilis.

### 3.6. Treatment Efficacy

A total of 92 PWH (86%) received treatment with BPG, 14 PWH (13.1%) were treated with doxycycline, and the case of neurosyphilis was treated with ceftriaxone. Treatment success, defined by adequate serologic response at the 12th month, was observed in 89 out of 92 PWH (96.7%) who received penicillin, as well as in 13 out of 14 PWH (92.9%) who were treated with doxycycline (Table 4). The remaining four cases were serological non-responders who did not achieve a fourfold decline in RPR titer. No treatment failure was observed. There was no statistically significant difference in effectiveness observed between the two treatments (*p* = 0.438, Fisher’s exact test). The treatment of neurosyphilis with ceftriaxone was also successful. No adverse drug effects were observed in any treatment.

## 4. Discussion

This study observed that nearly one in ten PWH engaging in risky sexual behaviors developed syphilis during a four-year monitoring period. Furthermore, approximately one-third of the study participants had experienced at least one syphilis episode in the past. This significant rate of syphilis diagnosis aligns with findings from other recent studies [25,26,27]. This trend may be attributed to an increased proportion of individuals engaging in unprotected sex due to reduced concerns about STIs, including HIV infection [18,20]. The shift toward viewing HIV infection as a manageable chronic condition has inadvertently led to decreased emphasis on safe sex among certain populations, resulting in higher rates of new HIV diagnoses and co-infections with syphilis [19,28].

STI surveillance data from the COVID-19 pandemic period must be interpreted with caution due to significant disruptions in access to public health services, particularly during lockdowns, which often affected routine preventive screening for STIs. Despite these challenges, a global increase in syphilis incidence was noted during the pandemic. While the CDC and other studies indicated a rise in syphilis cases as early as 2020 compared to 2019, data from the ECDC initially showed a decline in syphilis diagnoses in 2020, followed by a rapid increase in 2021 and 2022 [1,2,29,30,31]. Our study aligns with the findings of the ECDC report. In 2020, we observed an approximately 50% decline in the number of syphilis diagnoses compared to 2019, followed by a gradual increase over the subsequent two years, although it did not reach the levels of 2019. Importantly, outpatient care for PWH in our department remained largely unaffected during the COVID-19 period, as evidenced by patient turnout and the consistently high number of tests conducted. The decline in syphilis rates in 2020 may have been influenced by a significant portion of the Greek population adhering to pandemic prevention measures and reducing social contacts during the initial months of the COVID-19 period, a trend that waned in the following period.

We found that all cases of syphilis involved males, predominantly younger MSM. A notable proportion of these cases appeared to be reinfections during the study period. MSM and individuals with unknown transmission risk had a higher rate of syphilis diagnosis compared to heterosexuals and PWID. Furthermore, the incidence of previous syphilis infection and the likelihood of co-infection with syphilis at the time of HIV diagnosis was significantly higher among MSM than in the other transmission groups. MSM are disproportionately affected by STIs such as gonorrhea and primary and secondary syphilis [1,2]. Co-infection with HIV is prevalent among this group, with recent data in EE showing that 26% of MSM diagnosed with syphilis also had HIV [2]. This percentage is higher among individuals with primary and secondary syphilis. The factors associated with the rising incidence of syphilis among MSM include living with HIV, having a higher number of non-steady male partners engaging in condomless anal intercourse, longer intervals between STI screenings, involvement in sex work, and the use of PrEP for HIV prevention [2,17].

The prevalence of syphilis stages varies among different studies [32]. We noted that the majority of syphilis cases involved latent stages, with only one case presenting as neurosyphilis. The presence of asymptomatic syphilis should not offer reassurance to affected individuals; rather, it should raise significant concerns. If left untreated, latent syphilis can progress to tertiary syphilis or neurosyphilis [22]. From a public health perspective, pregnant women with latent syphilis can also transmit *Treponema pallidum* to their fetus, and individuals with early latent syphilis can unwittingly transmit the disease to sexual partners through recently healed lesions, thereby contributing to the community-wide transmission of syphilis [22,33]. Therefore, early detection and treatment of syphilis are crucial for preventing serious complications and reducing transmission rates.

Effective prevention strategies should focus on promoting regular STI screening, including syphilis, addressing barriers to condom use, providing comprehensive sexual health education, and ensuring access to PrEP and other prevention tools while also promoting safe sexual practices [1,34,35]. Identifying cases through enhanced screening of PWH who are at risk, along with incorporating syphilis testing into routine HIV clinical monitoring, is essential [1,34,35]. Addressing these factors comprehensively can help mitigate the rising incidence of syphilis within PWH.

We did not identify any significant correlations between incident syphilis cases and HIV-related characteristics, including the duration of HIV infection, the duration of ART, CD4+ T-cell count, and HIV viral load > 50 c/mL. Although there are studies suggesting that syphilis may increase viral load levels and decrease CD4+ T lymphocytes, the data are contradictory [36]. This is partly due to the lack of consensus among studies and also because these disruptions may be temporary [37,38]. It is more evident that ART improves the outcome of syphilis by reducing neurosyphilis episodes through immunological restoration and enhancing treatment effectiveness [9]. Since most PWH were receiving ART, this could partly explain the lower incidence of neurosyphilis in our study.

The treatment of choice for all stages of syphilis (excluding neurosyphilis) in PWH remains intramuscular BPG, with reported rates of serological response ranging from 70% to 95% in the international literature [22,32]. Alternative treatment options include doxycycline and ceftriaxone [22]. In our clinic, we typically treat syphilis with BPG. If penicillin is not available or if there is a history of documented allergy, we administer doxycycline. The majority of study participants (86%) received penicillin. A total of 96.3% of total syphilis cases (103 out of 107) exhibited a serological response 12 months after starting treatment. We found no statistically significant difference in effectiveness between BPG and doxycycline, suggesting that doxycycline is a viable alternative option. The need for new antimicrobial agents is an ongoing domain of discussion and research [15,16]. While penicillin remains highly effective for syphilis, the exploration of new treatments is motivated by factors such as penicillin shortages, patient inconvenience with multiple injections, and allergies [32]. There have been reports of penicillin-resistant strains of syphilis, although they are still relatively rare [39]. A recent systematic review and meta-analysis of 27 studies showed that monotherapy with penicillin did not outperform other monotherapies, such as doxycycline, ceftriaxone, and azithromycin, in terms of serological conversion [32]. This suggests a potential to reduce global reliance on penicillin, including for PWH, when it is unavailable. Likewise, since there is no vaccine for syphilis, post-exposure prophylaxis with doxycycline (Doxy-PEP) has been extensively studied in recent years, and its effectiveness has been demonstrated in numerous studies [40,41]. However, its use as an alternative to the currently recommended prophylactic treatment with penicillin within 90 days of exposure has not yet been widely adopted due to concerns about the potential impact on antimicrobial resistance and the microbiome [42,43].

This study has several limitations. Firstly, it is a single-center study that focuses solely on PWH, and there is no control group without HIV infection for comparison. Additionally, the participants were predominantly male PWH, reflecting the male-dominated composition of PWH monitored in our department. Therefore, our findings may not be generalizable across genders. The interval between syphilis testing varied among participants, and there were occasional dropouts during the study. Furthermore, individuals living with HIV can also undergo syphilis and STI testing outside of HIV care at other sexual health clinics, potentially resulting in an underestimation of the diagnosis rate of incident syphilis cases. It is possible that some cases of syphilis were missed due to false-negative RPR tests and the reduced sensitivity of the nontreponemal tests in defining incident syphilis cases. Similarly, some cases of asymptomatic neurosyphilis may have been missed because not all participants underwent a lumbar puncture. Finally, the high ratio of penicillin to doxycycline (6.7:1) in the treatment of syphilis, along with the small number of cases that received doxycycline, may potentially limit the conclusions when comparing the effectiveness between the two treatments.

Our study stands as one of the scarce inquiries into the prevalence and treatment response of syphilis among PWH in Greece. Further studies need to be conducted to provide useful data and promote public health.

## 5. Conclusions

In conclusion, our study underscores a concerning incidence of syphilis among PWH engaging in high-risk sexual behavior, particularly among younger MSM. Regular screening and comprehensive sexual health education are essential for early detection and treatment to prevent disease progression and transmission. BPG remains the preferred treatment option, but alternative regimens such as doxycycline have shown positive outcomes, highlighting the importance of flexibility in treatment approaches to accommodate penicillin shortages, allergies, and patient preferences.

## Figures and Tables

**Table 1:** Demographic and clinical characteristics of PWH at baseline (*n* = 991).

Characteristics	Result
Gender, *n* (%)	
Male	900 (90.8)
Female	91 (9.2)
Age in years, median (IQR)	41.3 (34.3–49.7)
Age in years, *n* (%)	
16–29	153 (15.4)
30–39	286 (28.9)
39–49	310 (31.3)
50–59	180 (18.2)
>60	62 (6.3)
Mode of transmission, *n* (%)	
MSM	641 (64.7)
Heterosexual	141(14.2)
PWID	122 (12.3)
MSM + PWID	4 (0.4)
Unknown/Other	83 (8.4)
Ethnicity, *n* (%)	
Greek	874 (88.2)
Other	117 (11.8)
Duration of HIV infection in years, median (IQR)	4.7 (0.3–10.3)
Duration of HIV infection in years, *n* (%)	
0–1	317 (32)
1–5	191 (19.3)
5–10	225 (22.7)
>10	258 (26)
Initial referral to IDU, *n* (%)	249 (25.1)
Duration of ART in years, median (IQR)	3.3 (0–8)
CD4+ T cell count, cell/mm^3^, median (IQR)	594 (391–814)
CD4+ T cell count < 200 cell/mm^3^, *n* (%)	90 (9.1)
HIV RNA < 50 c/mL, *n* (%)	704 (71)
History of syphilis, *n* (%)	320 (32.3)
MSM	265 (41.3)
Heterosexual	21 (14.9)
PWID	14 (11.5)
MSM + PWID	0 (0)
Unknown/Other	20 (24.1)

PWH, people with HIV; *n*, number; IQR, interquartile range; MSM, men who have sex with men; PWID, people who inject drugs; HIV, human immunodeficiency virus; IDU, infectious diseases unit; ART, antiretroviral therapy; CD4, cluster of differentiation 4.

**Table 2:** Syphilis diagnoses and syphilis testing by year.

Variable	Year				Total
	2019	2020	2021	2022	
Syphilis cases	35	18	25	29	107
Syphilis tests	398	410	496	592	1896
PWH tested for syphilis	362	384	395	532	1673

PWH, people with HIV.

**Table 3:** Characteristics of incident syphilis cases and bivariate and multivariable regression models for new syphilis diagnosis.

Characteristics	Incident syphilis (*n* = 107)	Crude OR	*p*	Adjusted OR	*p*
Age in years, median (IQR)	37.6 (31.8–46.8)	0.97 (0.96, 0.99)	0.004	0.98 (0.96, 1.003)	0.091
Age in years, *n*					
16–29	19	1.79 (0.98, 3.28)	0.06		
30–39	40	1.83 (1.11, 3.02)	0.019		
40–49	27	Ref			
50–59	17	1.1 (0.59, 2.04)	0.775		
>60	4	0.79 (0.27, 2.29)	0.66		
Ethnicity, *n*					
Greek	99	0.86 (0.41, 1.8)	0.679		
Other	8	Ref			
Mode of transmission, *n*					
MSM	97	5.12 (1.61, 16.29)	0.006	4.4 (1.36, 14.24)	0.013
Heterosexual	3	Ref		Ref	
PWID	2	0.90 (0.15, 5.43)	0.905	0.80 (0.13, 4.89)	0.807
MSM + PWID	0	(-)	(-)	(-)	(-)
Unknown/Other	5	2.263 (0.52, 9.46)	0.278	2.51 (0.59, 10.72)	0.215
Duration of HIV infection in years, median (IQR)	5.4 (2.5–9.8)	0.98 (0.95, 1.01)	0.185	0.97 (0.93, 1.01)	0.128
Duration of HIV infection in years, *n*					
0–1	21	1.39 (0.77, 2.51)	0.277		
1–5	28	1.46 (0.84, 2.53)	0.176		
5–10	32	1.39 (0.82, 2.37)	0.221		
>10	26	Ref			
Initial referral to IDU	17	1.21 (0.71, 2.01)	0.486		
Duration of ART in years, median (IQR)	4 (2.3–8.2)	0.98 (0.95, 1.02)	0.285		
CD4+ T cell count, cell/mm^3^, median (IQR)	656 (486–826)	1.00 (0.99–1.001)	0.969		
CD4+ T cell count <200 cell/mm^3^, *n*	3	0.47 (0.15, 1.49)	0.199	1.67 (0.50, 5.57)	0.403
HIV RNA > 50 c/mL, *n*	20	0.72 (0.44, 1.19)	0.204	0.64 (0.36, 1.13)	0.121
History of syphilis, *n*	59	0.77 (0.52, 1.13)	0.181	1.06 (0.71, 1.60)	0.768

OR, odds ratio; IQR, interquartile range; Ref, referent; MSM, men who have sex with men; PWID, people who inject drugs; HIV, human immunodeficiency virus; IDU, infectious diseases unit; ART, antiretroviral therapy; CD4, cluster of differentiation 4.

**Table 4:** Stages of incident syphilis cases and treatment efficiency.

Variable	*n* (%)
Stage of syphilis	
Early latent	25 (23.4)
Late latent	51 (47.7)
Primary	10 (9.3)
Secondary	20 (18.7)
Tertiary	0 (0)
Neurosyphilis	1 (0.9)
Treatment	
BPG	92 (86)
Doxycycline	14 (13.1)
Ceftriaxone	1 (0.9)
Treatment success	
BPG	89 (96.7)
Doxycycline	13 (92.9)
Ceftriaxone	1 (100)

BPG, benzathine penicillin G.

## Data Availability

All data generated in this study are included in this published article. Further inquiries can be directed to the corresponding author.
